# Hydrogen generation from NaBH_4_ solutions using a molecular mixed-valence nickel-substituted K_7_[Ni^III^Ni^II^(H_2_O)W_11_O_39_]·15H_2_O catalyst

**DOI:** 10.1039/d5ra04000k

**Published:** 2025-09-22

**Authors:** Yasemin Torlak, Ebru Halvacı, Bilge Akkoyun, Ayşenur Aygün, Fatih Sen

**Affiliations:** a Pamukkale University, Cal Vocational High School, Department of Agricultural and Livestock Production Cal Denizli 20700 Türkiye ytorlak@pau.edu.tr; b Sen Research Group, Biochemistry Department, Faculty of Arts and Science, Dumlupinar University Evliya Celebi Campus Kutahya 43100 Türkiye fatihsen1980@gmail.com

## Abstract

In the exploration for clean and sustainable energy, hydrogen has emerged as an important alternative energy carrier. In this study, K_7_[Ni^III^Ni^II^(H_2_O)W_111_O_39_]·15H_2_O (Ni-POM), a Keggin-type mixed valence polyoxometalate (POM) compound, was synthesised, and its catalytic effect on hydrogen production by hydrolysis of NaBH_4_ was investigated. The Ni-POM complex was characterised by UV-visible spectroscopy (UV-vis), FT-IR spectroscopy (FT-IR), X-ray diffraction (XRD), transmission electron microscopy (TEM), scanning electron microscopy (SEM), cyclic voltammetry (CV) and atomic force microscopy (AFM) methods. The characteristic absorption band observed at 265 nm in the UV-vis was attributed to ligand–metal charge transfer originating from the Ni(iii) centres. XRD analyses confirmed the presence of a highly crystalline Keggin-type structure. TEM images identified its nanosized morphology dispersed in the range of 5–15 nm. The effects of temperature, substrate and catalyst concentrations were determined, which gave highhydrogen production rate, a conversion frequency (TOF) of 806.38 s^−1^ and an activation energy (*E*_a_) of 22.26 kJ mol^−1^ at the optimum conditions for the Ni-POM catalytic activity studies. Results showed that Ni-POM is a stable and promising homogeneous catalyst capable of showing high catalytic activity in hydrogen production *via* hydrolysis of NaBH_4_. The findings suggest that mixed-valence POMs may open opportunities for innovative applications in hydrogen energy technologies.

## Abstract 2

Molecular mixed-valence nickel-substituted K_7_[Ni^III^Ni^II^(H_2_O)W_11_O_39_]·15H_2_O catalyst is used for hydrogen generation from NaBH_4_ solutions.

## Graphical abstract

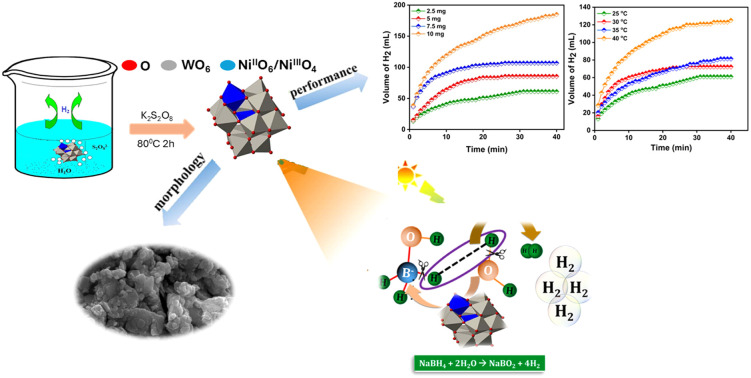

## 1. Introduction

Due to the rapid depletion of fossil fuels and the increasing negative impacts of environmental problems caused by the use of these resources for energy production, research on sustainable, environmentally friendly energy sources and technologies has intensified.^1^ Hydrogen (H_2_) stands out as the best alternative to fossil fuels due to its advantages such as environmental friendliness, high energy density and high efficiency when used in energy production. The continuous development of technology and the resulting increasing demand for energy accelerate the trend towards alternative energy sources, and hydrogen is one such energy source. In recent years, the number of studies on hydrogen production methods and their development has been increasing, with a focus on providing clean energy worldwide.^2^ Therefore, hydrogen is seen as the fuel of the future, and energy systems are being designed based on hydrogen.^3,4^ Hydrogen can also be produced from different sources and processes, such as the gasification of fossil fuels, pyrolysis of biomass or processes using fermentative microorganisms, electrolysis of water or photoelectrochemistry.^5–8^ The biggest obstacle to the widespread use of hydrogen is the storage and controlled release of hydrogen. Research on controlled hydrogen production has shown that hydrogen production by the hydrolysis of inorganic boron hydrides, especially ammonia borane (NH_3_BH_3_), lithium borohydride (LiBH_4_), sodium borohydride (NaBH_4_), and potassium borohydride (KBH_4_, PBH), is efficient, reliable and relatively cost-effective, and the recommended minimum value for hydrogen storage capacity is 6.5 wt%.^9–11^

Sodium borohydride (NaBH_4_) is the most investigated and widely used material among chemical hydrides. Pure NaBH_4_ has a hydrogen content of 10.8 wt%, while PBH has only 8.9 wt% hydrogen. It is important to note that the hydrogen storage capacity of metal borohydride compounds depends on the amount of water present in them. In the literature, studies in which hydrogen is obtained catalytically from an aqueous solution of basic NaBH_4_ are predominant. The major disadvantage of this method is that the hydrolysis by-product sodium metaborate (NaBO_2_) covers the catalyst surface and causes deactivation of the catalyst.^12,13^ In non-catalytic systems, NaBH_4_ hydrolysis is possible with steam at 110–150 °C. High yields of hydrogen can be obtained by this method.^14^

The release of 4 moles of H_2_ per mole of NaBH_4_, although at low temperature and pressure, occurs *via* hydrolysis dehydrogenation in aqueous solution.^13^ However, it is possible to produce hydrogen in high purity and efficiently, provided that the appropriate catalyst is used to accelerate the reaction kinetics of the hydrolysis of metal borohydride compounds. For hydrolysis to occur spontaneously without a catalyst and to reach maximum efficiency, suitable catalysts are needed. Many metal-based catalysts, such as noble,^15–17^ non-noble^18,19^ and alloy-based catalysts, are used to achieve these requirements.^20^

1 NaBH_4_ + (2 + *x*)H_2_O → NaBO_2_·*x*H_2_O + 4H_2_

As can be seen from eqn (1), 2 moles of the hydrogen produced are supplied from metal borohydride (MeBH_4_) and the remaining 2 moles from water.^21,22^ There are many catalyst preparation methods reported in the literature. There are different techniques for the formation of a homogeneous thin film of nanosize, such as the magnetron sputtering technique and low loading amounts on the desired surface.^22,23^

The catalytic partial oxidation process, which is an efficient process for hydrogen production, depends on the type, activity, selectivity and stability of the catalysts used to realize the catalytic partial oxidation process to obtain the target amount of hydrogen (with 99% H_2_ selectivity). Considering that the most suitable catalyst system is Ni-based catalysts, the distribution of these catalysts on the support, prevention of agglomeration and morphology control will greatly increase the efficiency of the products. The nanosize of these synthesized particles will lead to an increase in the catalyst surface area and will provide physical and chemical superiority, which will increase the activity of the partial oxidation reaction and its selectivity in the direction of products (CO and H_2_).

Nano-sized nickel compounds have attracted considerable attention in recent years as they can be used in catalytic reactions, magnetic materials, fuel cells and batteries, electronics, optics and many other fields. Nickel is resistant to external influences due to its paramagnetic properties and is used for electrolytic plating of objects. Nickel is classified as a ferromagnetic material exhibiting strong magnetic properties. This is due to its atomic structure, which allows unpaired electrons in its d-orbital to align when subjected to a magnetic field. This alignment creates a net magnetic moment, giving nickel recognizable magnetic behavior. Nickel's distinct advantage lies in its corrosion resistance and workability, which make it a popular choice in specialty alloys and industrial applications requiring magnetic properties. Nickel exhibits strong magnetic properties but has a lower saturation magnetization than iron and a lower Curie temperature (354 °C or 669 °F) than other metals. Its advantage lies in its durability and resistance to corrosion. Nickel maintains its magnetism up to 627 kelvin (354 °C), its Curie temperature, beyond which it transitions to paramagnetic behavior. It is also preferred as a catalyst in hydrogenation reactions. The size of nickel is important when used for catalytic effect, and nanometer-sized catalysts are generally preferred. In addition, nickel is used as the inner electrode material in multilayer ceramic capacitors, taking advantage of its good electrical conductivity, high melting point and low cost. In order to achieve as high a capacitance as possible in these capacitors, it is desirable that the nickel layer used as the active layer is both very thin and non-agglomerated, consisting of as small a powder as possible.^24,25^

Metal-oxide or metal/organic structures are multifunctional organic or multicomponent compounds with a three-dimensional framework enclosing metal ions or small metal-containing clusters in uniform interconnected pores.^26,27^ Values in the range of 1500–3000 m^2^ g^−1^ are quite common, but even values higher than 5000 m^2^ g^−1^ for some metal-oxide compounds have been reported, indicating a very large surface area and pore volume.^28^ In addition to these properties, their wide range of chemical compositions, three-dimensional crystal structure and superior adsorption properties, in contrast to the most well-known activated carbons, give the material a significant potential for hydrogen storage. In carbon-based compounds, the surface area and micropores are approximately proportional to the volume. Since hydrogen uptake is usually maximum in these structures at 77 K, research has been developing faster compared to other hydrogen storage materials.^29–35^

POMs are compounds with multi-electron reduction ability, high charge storage capacity and great potential for oxidation degradation. These high oxidation states (*i.e.*, W^VI^, Mo^V,VI^, and V^IV,V^) originate from being composed of transition metals. Moreover, these negatively charged discrete polynuclear transition metal-oxide anionic clusters offer many structural and compositional variations for different nanotechnological applications.^36–39^ Reversible multi-electron conversion in POMs without a structural change has recently attracted the attention of researchers due to its significant potential as a catalyst or redox mediator, the possibility of operation at low temperatures, and the reusability of these materials as catalysts.^40,41^ Although noble metal-based catalysts such as Ru and Pt show high activity, their availability and high cost have led to the investigation of their further use as a nickel-based transition metal catalyst, which is more cost-effective than noble metals.^42,43^ Recently, several studies on the use of nickel-based catalysts to overcome the lower catalytic activity compared to noble metal catalysts and morphological studies to increase the surface area are still being actively carried out.^44^

Recently, many non-precious transition metal-based materials such as sulfides, selenides, phosphides, nitrides and carbides have been presented as a suitable alternative to noble metal-based electrocatalysts. Moreover, these catalysts have been extensively investigated as catalysts in hydrogen applications due to their low cost, ease of use, abundant availability, activity, stability and durability. Among these metal compounds, nickel is considered to be the most suitable and therefore the most widely used for hydrogen production reactions due to its high stability even at high pH values, low cost, and ease of application.^45,46^

Ni-POM compounds have attracted much attention due to their outstanding properties, such as tunable band gap, high surface area and enhanced catalytic activity, in various applications in sustainable energy, environmental remediation, and recently, especially in hydrogen technologies.^44^ They improve energy storage in different electronic devices such as solar cells, batteries, and supercapacitors, and increase stability and improve solar cell efficiency. POMs enable photocatalytic water splitting and easily catalyze the conversion of protons and electrons to hydrogen gas, reducing the activation energy required for electrochemical reactions to occur. In this way, they can function as effective photocatalysts in the breakdown of water and produce hydrogen as a clean and renewable energy carrier.^47–50^

In this study, Keggin-type mixed valence-nickel substituted K_7_[Ni^III^Ni^II^(H_2_O)W_11_O_39_]·15H_2_O (Ni-POM) polyoxoanions were synthesised and structurally confirmed by various techniques, such as UV-vis, FT-IR, CV, SEM, XRD, TEM, EDX, and TGA. Here, we focus on the catalytic studies of nickel-substituted polyoxometalate as a homogeneous catalyst. The Ni-POM compound, which also contains a mixed-valence transition metal in its structure, has advantageous properties such as electron transfer transition, larger surface area and surface energy. This makes it have better electron transfer ability than nickel-based nanostructures. Therefore, in this study, charge transfer, morphological structure and catalytic activity studies of the Ni-POM catalyst are discussed in detail.

## 2. Experimental

### 2.1. Materials and apparatus

Acetic acid (≥99%), sodium borohydride (NaBH_4_, 96%), sodium tungstate (Na_2_WO_4_), nickel(ii) acetate tetrahydrate (Ni(OCOCH_3_)_2_·4H_2_O), potassium persulfate (K_2_S_2_O_8_), glacial acetic (CH_3_COOH), methanol (CH_3_OH), and all chemicals used in the experiments were purchased from Sigma Aldrich. All chemicals were purchased from commercial companies and used without further purification.

### 2.2. Material characterization

The crystalline structure of the sample was characterized using a PANalytical powder diffractometer (Model: Empyrean) with CuKα radiation of *λ* = 1.54056 Å, operating at 45 kV and 30 mA. FT-IR spectra were recorded on a PerkinElmer 2000 model instrument. Similarly, UV-vis spectra were recorded with a Shimadzu UV-1800 model instrument. Surface morphology was characterized using AFM, NT-MDT Ntegra model device. Morphological characterization of the Ni-POM compound in the powder form was carried out with a JEOL-7100 model scanning electron microscope (SEM) and JEOL JEM-1400 Plus model transmission electron microscope and through energy dispersive X-ray spectroscopy (EDX). Thermogravimetric analysis (TGA) measurements were carried out using a Mettler Toledo TGA/DSC2 Star System under an Ar mass flow rate of 20 sccm, from room temperature to 1000 °C with a heating rate of 10 °C min^−1^. The test sample of the compound weighed approximately 4.2130 mg. The return to ambient temperature (25 °C) from the 1000 °C at the end of the analysis was achieved with a negative temperature ramp of 33 °C min^−1^. All electrochemical experiments were conducted in a three-electrode electrochemical cell on a workstation of Compact Stat (Ivium Technologies B.V., Netherlands). The graphite rod (*d* = 3 mm, *A* = 0.0707 cm^−2^) was used as the working electrode, a platinum wire as the auxiliary/counter electrode (length = 5 cm, *d* = 0.5 mm) and Ag/AgCl (3 M KCl, *E* = 0.223 V ± 0.13 mV at 25 °C *vs.* SHE) as the reference electrode. The graphite rod was polished with 0.3 μm Al_2_O_3_ powder and sonicated in water for about 30 seconds after the polishing step. Finally, the working electrode was washed with ethanol and then dried with a high-purity nitrogen stream before use. To generate the voltammogram, the potential in the range of −1.0 to 1.5 V at a scan rate of 100 mV s^−1^ was applied to the graphite rod electrode modified with the Ni-POM compound using cyclic voltammetry.

### 2.3. Synthesis of K_7_[Ni^III^Ni^II^(H_2_O)W_11_O_39_]·15H_2_O (Ni-POM)

**Scheme 1:**
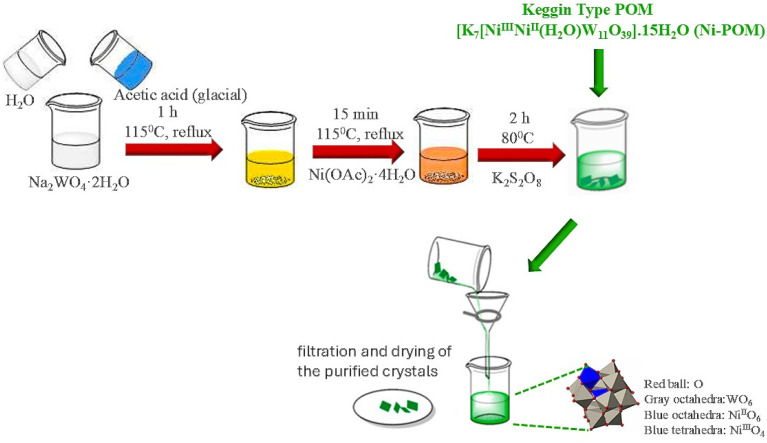
Illustration of the synthesis procedure of Ni-POM.

The synthesis of Ni-POM was performed in one stage as reported in our previous studies.^51,52^ Firstly, 40 g of sodium tungstate was dissolved in 80 mL of water. After the dissolution process was completed, the pH of this solution was adjusted between 6.5 and 7.5 by adding 20 mL of glacial acetic acid dropwise. The mixture was heated to reflux at 115 °C for one hour. Next, a solution containing 5% Ni(OAc)_2_·4H_2_O in 70 mL of water was added dropwise to this system while stirring. After adding this solution, the mixture was heated to reflux at 115 °C for two hours and then filtered while still hot to remove any traces of insoluble matter. The temperature of this solution was then adjusted to 80 °C. 28 g of K_2_S_2_O_8_ was added gradually to the solution. A large amount of gas was instantly formed during the addition. Once the addition was complete, the solution was adjusted again to a temperature of 115 °C. Finally, the color changed from light brown to dark green. The resulting mixture was boiled for 15 more minutes. This mixture was then cooled in an ice bath, and dark green precipitate formed rapidly. Then, the resulting dark green crystals were precipitated, filtered off and washed with distilled water. Finally, it was recrystallized with 50% methanol solution and then dried at room temperature. Yield: 5.05 g (15%) (Scheme 1).^52,53^

### 2.4. Catalytic activity study

In this study, catalytic activity tests were conducted using the precursor compound NaBH_4_ for hydrogen production *via* the water displacement reaction. The experiments were carried out in glass reactors equipped with magnetic stirrers under constant temperature control (±0.5 °C). The reaction medium was prepared by adding different amounts of catalysts (2.5 mg, 5.0 mg, 7.5 mg, and 10 mg) dissolved in 4 mL of deionised water. Furthermore, to investigate the effect of NaBH_4_ concentration on the reaction kinetics, experiments were conducted at concentrations of 300 mM, 350 mM, 400 mM, and 450 mM. Similarly, control experiments were repeated at 25 °C, 30 °C, 35 °C, and 40 °C to investigate temperature dependence.

## 3. Results and discussion

### 3.1. Characterization of Ni-POM

**Fig. 1:**
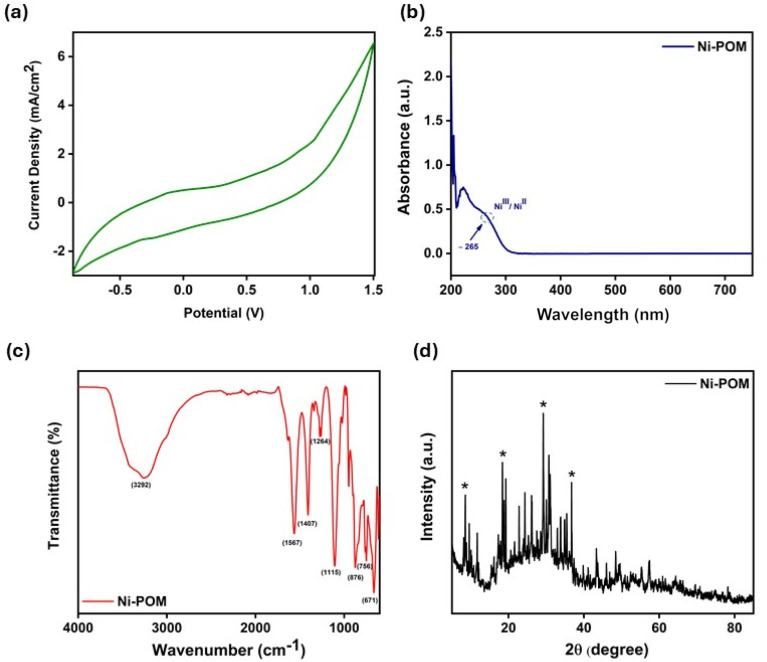
(a) CV of Ni-POM (10^−3^ M) in acetonitrile on graphite electrode using Bu_4_NBF_4_ (10^−1^ M) as the support electrolyte. (b) UV-spectrum of Ni-POM in water. (c) FT-IR spectra of Ni-POM. (d) XRD patterns of Ni-POM.

CV is a powerful and popular electrochemical technique widely used to investigate the reduction and oxidation of metal centres in POMs and to evaluate oxidation levels. Voltammetric electrochemical characterisations of Ni-containing POMs have been carried out to analyze the spectral and structural changes accompanying electron transfer. The electrochemical characteristics of Keggin-type POMs in acetonitrile were investigated by cyclic voltammetry. The observation of two peaks shown by the metal of interest indicates that they are due to Ni(iii)/Ni(ii) oxidation. Moreover, the potential values are in the positive region, which proves that the metal in the low oxidation state is strongly bound to the ligand.^54^Fig. 1a shows two oxidation peaks for Ni-POM Ni(iii)/Ni(ii) around 0.645 and 1.156, reduction peaks around 0.524 and 0.972, corresponding to the CV of the structure with a central Ni valence of +2 in 0.1 M TBABF_4_ solution.^55^

Absorbance spectra of POM solutions were obtained in water as the solvent using a UV-vis spectrometer. When the absorbance graph of Ni-POM (Fig. 1b) is analysed, absorption is observed at 265 nm wavelength. UV-vis spectroscopic investigations of Ni-POM (Fig. 1b) (H_2_O, pH: 7.0) show an absorption at 265 nm.^53,56,57^ Since the Ni-POM compound contains both Ni(ii) and Ni(iii) mixed valence centres in its structure, the band at 265 nm is specifically attributed to the d–d transition of the Ni(iii) centre and the ligand–metal charge transfer (LMCT) band (Fig. 1b).^58^ This Ni(iii) valence in the structure also contributes to the stability of the Keggin-type polyanionic structure and the increase in hydrogen production efficiency.^59^

FTIR spectra of Ni-POM are characterised in the range 400–4000 cm^−1^. The FT-IR spectrum of the Ni-POM compound confirmed the presence of a heteropolyanion Keggin-type structure with a central Ni(iii)O_4_ tetrahedron. FT-IR spectra of Ni-POM are given in Fig. 1c. The characteristic peaks of Ni–O bending stretching modes at 1115 cm^−1^ (sh) and 876 cm^−1^ (s) are attributed to the tetrahedral sites of oxygen–metal ions such as W–O_b_ (corner-sharing) and W–O_d_ (terminal).^52^ Moreover, the peaks of Ni–O bending stretching modes at 671 cm^−1^ (s), 756 cm^−1^ (m), 1264 cm^−1^ (w), 1407 cm^−1^ (w) and 1567 cm^−1^ (w) point to oxygen–metal ions in tetrahedral sites.^60^ The presence of water in the coordination site is also evident from the broad spectrum intensity at 3292 cm^−1^.^61^

XRD analysis provides very important information on the crystal structure of POMs in powder form, in qualitative and quantitative analysis, and in the characterization of the morphological properties of the internal structure. As seen in Fig. 1d, the XRD pattern of Ni-POM showed intense peaks in the angle range of 15° to 40°, confirming that it is in the native form crystal structure. When the POMs synthesized in the present study are compared with the POM compounds synthesized in literature studies, it is seen that they have similar structures. The presence of mixed valence transition metals in the POM compounds with Keggin structure is closely related to the vacancy crystallinity of these sites.^60,62,63^

**Fig. 2:**
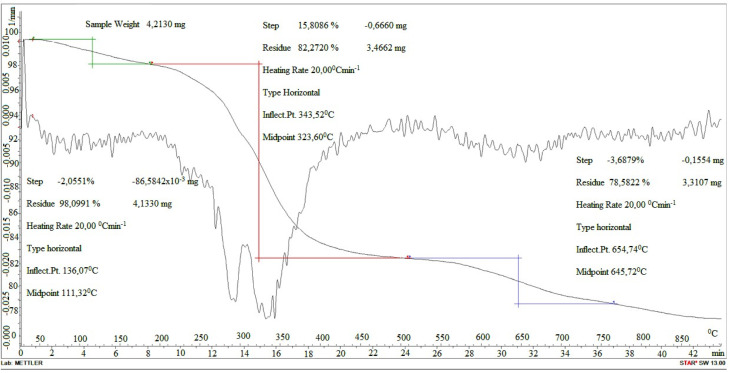
TGA thermogram of Ni-POM.

Fig. 2 shows the thermogram of Ni-POM. Thermal behavior and thermal stability of Ni-POM were studied by TGA, which exhibits weight loss steps in the temperature range of 25–800 °C. In this investigation, heating rates were suitably controlled at 10 °C min^−1^ under N_2_ atmosphere, and the weight loss was measured from ambient temperature up to 1000 °C. The thermogram of Ni-POM shows weight loss, which starts at room temperature due to dehydration, corresponding to the loss of 15 crystal water molecules. The thermal degradation profile obtained for Ni-POM is in agreement with the results obtained from other polyoxometalate compounds containing similar organic cations in the literature.^65–67^

Cationic salts resulted in the complete loss of the organic fraction with the formation of mixed metal oxides, with relatively good agreement between experimentally observed and theoretically derived mass losses upon heating to 450 °C.

**Fig. 3:**
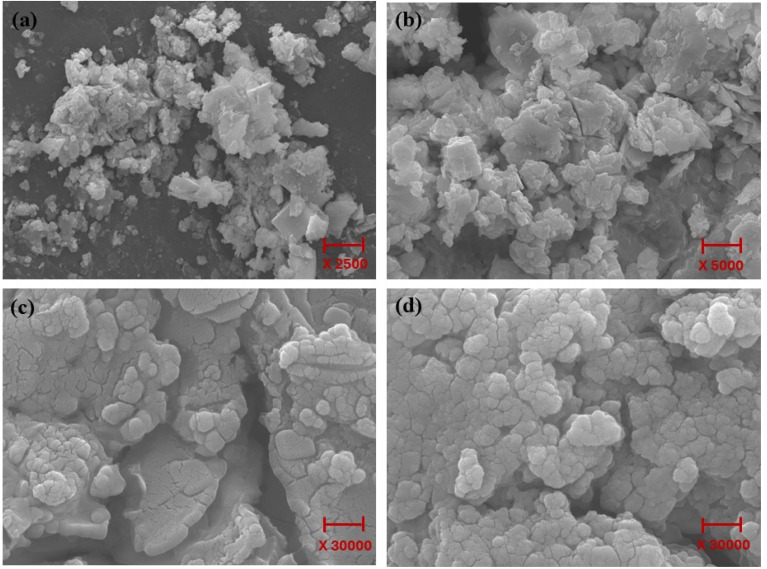
SEM images of Ni-POM at (a) 2500×, (b) 5000× and (c and d) 30 000× magnifications.

Scanning electron microscopy (SEM) is one of the most versatile and widely used tools for studying the surface morphology and topographical properties of POMs. SEM images of the Ni-POM structure are shown in Fig. 3a–d. When these images are evaluated, it can be clearly observed that POM nanoparticles show homogeneous and interconnected networks that interact with each other. In addition to the individual characteristics of the active components and support material of the prepared catalyst, it is also important that these components work in harmony to exhibit superior properties. Several parameters, such as the compatibility of the active metals with the surface, their homogeneous distribution, size and shape, and their permanence on the support, affect the success of the catalyst.^64^ Structures synthesized at nanoscale, representing a blend of composition- and functionality-based properties, are widely used as self-healing, bioelectronic, programmable, lipid-based, protein-based, and antibacterial materials.^65^ The large and small pores seen on the POM in SEM photographs allow the movement of reactants and products during the reaction. Therefore, it should be expected that the diffusion resistance of the POM catalyst is more limited and that this situation contributes to the hydrogen production rate. In hydrogen production, the aim is to keep the active particle sizes as small as possible in catalysts. In this way, more active surface contact opportunities and less dwell times are provided for the molecules to react. In short, the reaction rate is increased.^66^ The small size of the particles can cause more edges and corners on the catalyst particles, molecular irregularities and different ionic charges. The extreme conditions made possible by these irregularities and differences prepare the ground for the reactions to occur easily.^67^

**Fig. 4:**
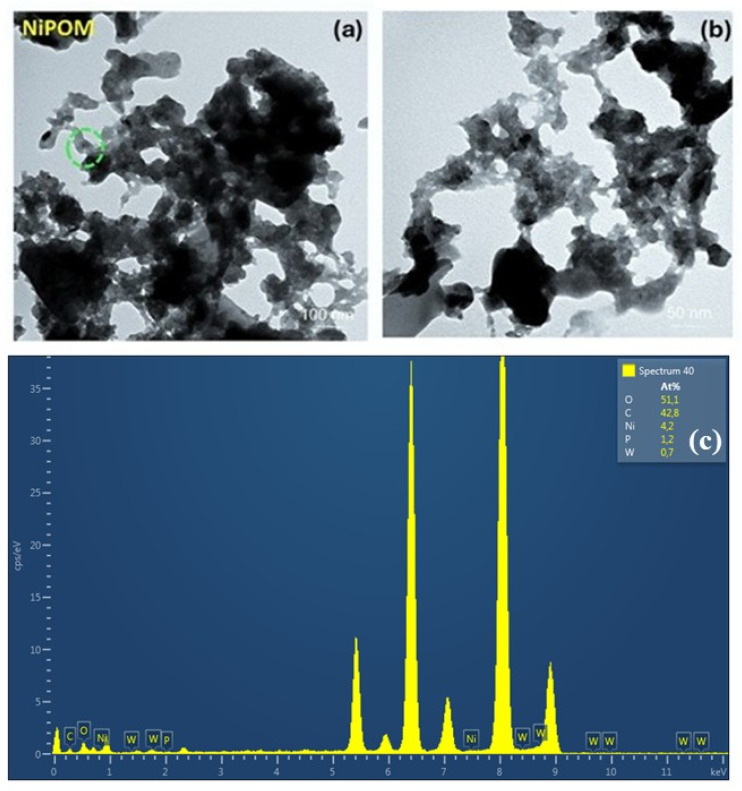
TEM images with different scales (a) 100 nm and (b) 50 nm ; (c) EDX analysis of Ni-POM.

Transmission electron microscopy (TEM) images presented in Fig. 4a and b provide the opportunity to examine the morphological properties and particle size distribution of the synthesised Ni-POM structure in detail. TEM analyses reveal that Ni-POM particles generally have a spherical morphology and monodisperse distribution. However, a tendency for agglomeration between particles is observed in some regions. This can be attributed to the tendency of small nanoparticles to approach each other, especially under the influence of van der Waals attraction forces. The observed structures show that particles of size 25 nm and above come together to form larger structures. In general, the size of POM particles was in the range of 50–100 nm, and agglomerated particles were also detected within this size range (>50 nm). Bright field TEM images show that the particles exhibit a well-dispersed polycrystalline structure on the surface; however, localised regions of agglomeration are evident in some surface areas. EDX analysis was carried out to investigate the distribution of desired elements in the K_7_[Ni^III^Ni^II^(H_2_O)W_11_O_39_]·15H_2_O (Ni-POM) compound (Fig. 4c). Also, it shows the chemical composition of the synthesized Ni-POM. EDX elemental analysis in Fig. 4 supports the presence of nickel and tungsten in these regions. The presence of P, C, N, O and Ni elements (elemental analysis calculated (%); P: 1.2, C: 42.8, O: 51.1, Ni: 4.20, W: 0.7) in this analysis confirms the successful synthesis of the Ni-POM compound, which is in agreement with the FT-IR and TGA results.^68,69^

**Fig. 5:**
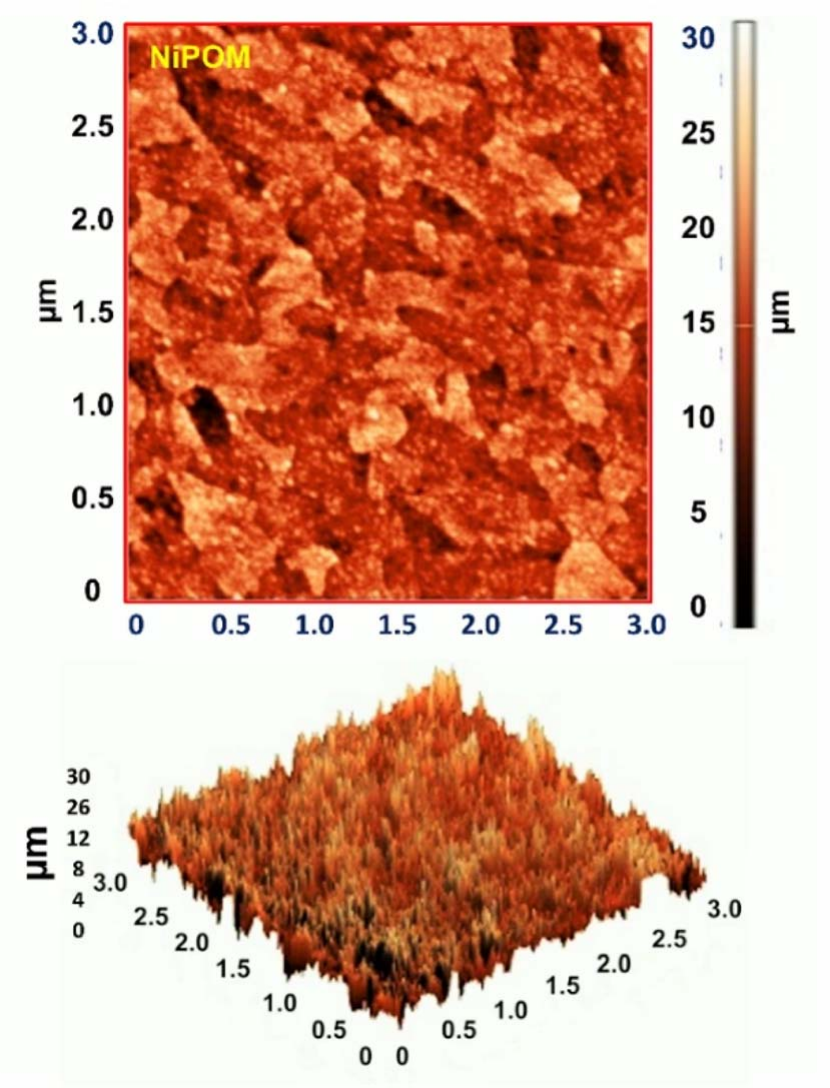
Two-dimensional (2D) and three-dimensional (3D) AFM images of Ni-POM.

In order to visualize the enhanced surface roughness of this compound containing mixed-valence transition metals, representative AFM images obtained under similar magnifications were studied. As shown in Fig. 5, the 2D and 3D topographies of the synthesized Ni-POM compound were characterized by AFM using nanoscale profiling. Two-dimensional, three-dimensional and topographic images show that Ni-POM is spherical. The particle size distribution was measured by AFM characterization. As a result of this measurement, it was determined that Ni-POM is a crystalline compound with dimensions between approximately 1–2 nm. In addition, upon analyzing the AFM images, an increase in dimensions of 20–30 nm was also observed. The results of the analysis obtained are consistent with the studies in the literature.^51,52^Fig. 5 shows the AFM images of the POMs. The root mean square surface roughness values and the average roughness increased from 2.08 and 1.63 nm for Ni-POM. In addition to this information, the structure of the POM surface, as observed by a 3D AFM image, shows that the distribution of clusters is almost uniform.

### 3.2. Catalytic activity

The economic sustainability of a process in catalytic activity studies is prioritised to have appropriate costs for each component used. In addition, catalysts used in chemical processes can increase the cost of the process depending on the content and production conditions. For this reason, the catalysts should be prepared from affordable materials. In addition, most of the active structures used in catalysts consist of noble metals.^70–72^ The high cost of noble metals, coupled with the limited lifetime of catalysts, can lead to an economic dilemma. Hybrid catalyst trials show that the presence of a certain amount of noble metals in the catalyst structure provides sufficient activity and increasing the amount does not improve or even reduce the activity. For this reason, catalysts produced with transition elements used alone and POM compounds, which are the most successful among these metals, both provide the desired activity and keep catalyst costs at an affordable level. This POM catalyst, containing a certain amount, rapidly initiates the hydrolysis process. Considering the total hydrogen production, catalysts containing a certain amount of POM by mass show superior properties, and it is clear that they can perform the hydrolysis process at desired rates. The positive effect of the relationship between catalyst structures containing mixed-valence metals on activity is observed.

**Fig. 6:**
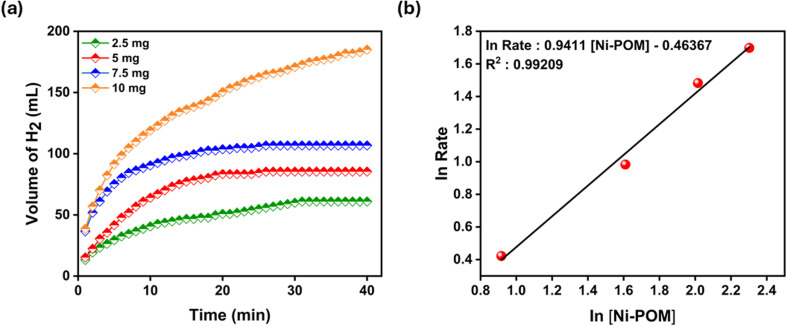
(a) H_2_ production volume versus time graph in the presence of different amounts of catalysts [NaBH_4_ concentration 300 mM, 25 °C] and (b) logarithmic ratio of hydrogen release *versus* logarithmic concentration of Ni-POM for NaBH_4_ hydrolysis.

NaBH_4_ hydrolysis reaction was carried out to determine the catalytic activity of Ni-POM, as shown in Fig. 6, and the amount of catalyst providing the highest catalytic activity.^73–75^ In this process, the amount of catalyst was determined as 2.5 mg, 5 mg, 7.5 mg, and 10 mg and analysed in terms of hydrogen release rate, while other parameters were kept constant (25 °C, 300 mM NaBH_4_). According to the results obtained, it was observed that Ni-POM was active at very low amounts (2.5 mg). The hydrogen release rate is shown in Fig. 6a, and in Fig. 6b, the hydrogen release rate was measured by calculating from the linear part of the graphs. It is plotted against the initial catalyst concentration on a logarithmic scale. It is observed that the hydrolysis of NaBH_4_ depends on the first-order catalyst concentration with a slope of 0.99209.

### 3.3. Effect of NaBH_4_ concentration

The decomposition of NaBH_4_ produces hydrogen and NaBO_2_ as a by-product. NaBH_4_ contains 2 mol of hydrogen, while 4 mol of hydrogen are produced by the reaction. The additional hydrogen gained is due to the decomposition of water. Kinetic studies were carried out at different NaBH_4_ concentrations, constant catalyst amounts and temperatures. Measurements were carried out under atmospheric conditions, and the gas produced was assumed to be ideal. The hydrogen produced as a result of the reaction was collected in an inverted burette, and the volume was recorded *versus* time. In order to provide a better understanding of the experimental results expressed graphically, the hydrogen production rate results were also taken into account.

**Fig. 7:**
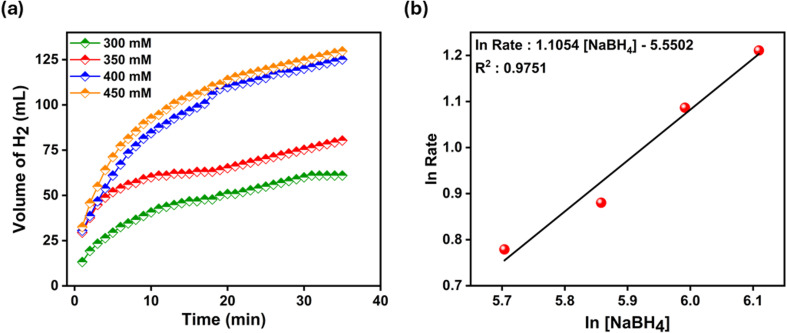
(a) H_2_ production volume versus time graph at different NaBH_4_ concentrations [Ni-POM 2.5 mg, 25 °C] and (b) logarithmic ratio of hydrogen release *versus* logarithmic concentration of NaBH_4_ for NaBH_4_ hydrolysis.

To understand the effect of NaBH_4_ amount on the hydrolysis reaction, NaBH_4_ at concentrations of 300 mM, 350 mM, 400 mM and 450 mM was used. The compiled results for the catalysts are shown in Fig. 7. An increase in gas volume was observed with increasing the amount of NaBH_4_ in the hydrolysis solution. At low NaBH_4_ values, the reaction ended before it had time to accelerate, and the hydrogen production rate values for 300 mM and 350 mM NaBH_4_ were low due to the low solution concentration. In the experiments with 400 mM and 450 mM NaBH_4_ concentrations, it was observed that the hydrogen production rates were close to each other, and the hydrolysis rate increased with the amount of NaBH_4_ used. This increase is also noticeable in the graph in Fig. 7a. The increase in solution viscosity at high NaBH_4_ concentrations, the increase in the amount of by-product NaBO_2_ as the reaction progresses and the easier access to the active sites should be considered as the reason for this increase.^74,76,77^ When the initial velocities for varying NaBH_4_ amounts are considered in Fig. 7b, it is seen that the hydrolysis reaction is independent of the NaBH_4_ amount and is of zero order.

### 3.4. Effect of hydrolysis temperature

**Fig. 8:**
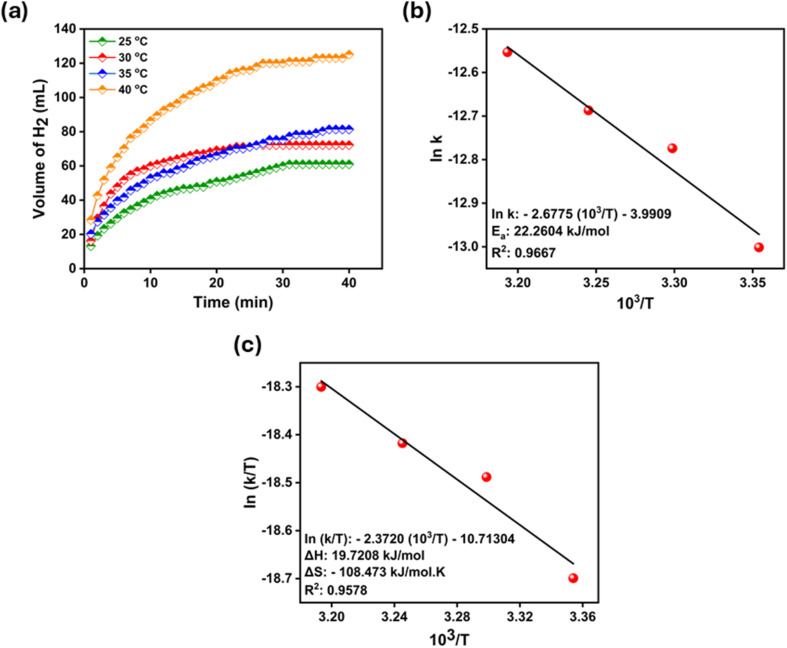
(a) H_2_ production volume versus time graph depending upon temperature effect on. (b) Arrhenius plot and (c) Eyring plot of the Ni-POM catalyst for NaBH_4_ hydrolysis.

Experiments were carried out at different temperatures (25 °C, 30 °C, 35 °C, and 40 °C), keeping the catalyst (2.5 mg) and NaBH_4_ (300 mM) concentrations constant to analyse the effect of temperature on NaBH_4_ hydrolysis.^71^Fig. 8a shows the plot of moles of hydrogen per mole of NaBH_4_*versus* time at temperatures between 25 °C and 40 °C. The results obtained show that there is a linear relationship between temperature increase and hydrogen release.

The rate constants were calculated from the linear sections of the graphs in the figure. The activation parameters in Fig. 8b and c were also calculated using rate constants (*k*). Calculations were made by plotting ln *k* − (10^3^/*T*) for Arrhenius and ln(*k*/*T*) − (10^3^/*T*) for Eyring plots. According to these calculations, the *E*_a_, Δ*H* and Δ*S* values of the catalyst were found to be 22.26 kJ mol^−1^, 19.72 kJ mol^−1^ and −108.473 kJ mol^−1^ K^−1^, respectively. The obtained *E*_a_ value is significantly lower than the values of conventional catalysts used for hydrogen produced by NaBH_4_ hydrolysis (Table 1). According to these results, it was observed that the use of Ni-POM provided significant catalytic activation for hydrogen production *via* NaBH_4_ hydrolysis.

**Table 1:** Comparison of the hydrogen generation rate (HGR), activation energy (*E*_a_) and TOF of different Ni-based catalysts in the literature with those of the Ni-POM catalyst

Catalyst	Solvent	HGR (mL min^−1^ g^−1^)	TOF (s^−1^)	Activation energy (kJ mol^−1^)	Ref.
Ni–Co/r-GO	Hydrolysis	1280	—	55.12	80
Ni(0)	Hydrolysis	—	—	41.76	81
NiMo/Cu	Hydrolysis	6.48	—	60.49	82
Ni–B–Cr_0.8_@RH	Hydrolysis	1428	—	50.64	83
Ni nanoparticles	Hydrolysis	1000	—	69.76	84
Ni–B	Hydrolysis	333	—	61.84	85
Ni-POM	Hydrolysis	610.2	806.38	22.26	**This work**

### 3.5. Reusability tests of Ni-POM during NaBH_4_ hydrolysis

In this study, the Ni-POM catalyst was also tested for reusability in the hydrolysis of NaBH_4_. Reusability tests have not traditionally been performed for the homogeneous nature of the Ni-POM catalyst. Homogeneous catalysts, which, like heterogeneous catalysts, are not easily separated from the reaction medium and can be subjected to multiple reuse cycles, remain dissolved in the reaction solution. This makes it difficult for them to be physically recovered and subsequently reused. Therefore, the concept of reusability is less applicable to homogeneous systems, as it does not provide reliable information about catalyst stability or performance in the long term. Instead, an approach to the assessment of the catalytic stability of Ni-POM has been taken, in which catalytic activity can be monitored under time-varying reaction conditions instead of standard recycling protocols.^74,78,79^

Table 1 compares the hydrogen generation rates (HGR) and activation energy values of various catalysts in the hydrogen generation reaction in the same solvent. The Ni-POM catalyst synthesised in this study exhibits an HGR value of 610.2 mL min^−1^ g^−1^ and an activation energy as low as 22.26 kJ mol^−1^. Compared to other catalysts in the literature, the decrease in the activation energy of Ni-POM is evident. For example, Ni–Co/r-GO (55.12 kJ mol^−1^), NiMo/Cu (60.49 kJ mol^−1^), Ni–B–Cr_0_._8_@RH (50.64 kJ mol^−1^) and Ni nanoparticles (69.76 kJ mol^−1^) have quite high activation energy values. These statistics imply that Ni-POM requires a lower energy barrier to initiate the reaction, *i.e.*, it shows greater energy efficiency. These findings reveal that Ni-POM requires a lower energy barrier to initiate the hydrogen production reaction, which provides a significant advantage in terms of energy efficiency. In terms of HGR performance, the value of 610.2 mL min^−1^ g^−1^ of Ni-POM, although relatively lower than some catalysts in the literature, is quite competitive compared to, for example, Ni–B (333 mL min^−1^ g^−1^) and NiMo/Cu (6.48 mL min^−1^ g^−1^) catalysts. Moreover, compared to catalysts with high HGR values such as Ni–Co/r-GO (1280 mL min^−1^ g^−1^) and Ni–B–Cr_0_._8_@RH (1428 mL min^−1^ g^−1^), the low activation energy of Ni-POM provides a significant advantage in terms of energy efficiency. This indicates that Ni-POM is a potential alternative for sustainable catalytic processes by minimising the energy consumption. Furthermore, only the TOF (Turnover Frequency) value for the Ni-POM catalyst is reported in the table. This information is an indication that Ni-POM offers high performance in terms of surface activity and catalytic conversion capacity. As a result, Ni-POM catalyst provides energy efficiency with low activation energy, while providing results that can compete with many studies in the literature in terms of HGR performance. These properties make Ni-POM stand out as an effective and sustainable catalyst for hydrogen production.

## 4. Conclusion

Hydrogen (H_2_), a renewable energy source, is considered a promising energy carrier for future applications due to its environmentally friendly fuel properties and high energy density. Catalytic hydrolysis of NaBH_4_ is an important process for the production of hydrogen H_2_. This innovative methodology, particularly using transition metal-based catalysts as direct catalysts, has gained widespread acceptance due to its cost-effectiveness and environmentally friendly approach. In this study, K_7_[Ni^III^Ni^II^(H_2_O)W_111_O_39_]·15H_2_O (Ni-POM), a Keggin-type mixed valence polyoxometalate as a catalyst, was successfully synthesized, and its catalytic performance on hydrogen production by NaBH_4_ hydrolysis in aqueous media was evaluated in detail. As a result of structural characterisations, it was determined that Ni-POM retains its crystalline structure and its nanoscopic morphology, which contribute positively to the catalytic activity. As a result of kinetic studies, very impressive catalytic parameters such as conversion frequency (TOF) of 806.38 s^−1^ and activation energy (*E*_a_) of 22.26 kJ mol^−1^ were found. The catalytic tests confirmed that Ni-POM showed an excellent performance for hydrogen production. The hydrogen production rate increased significantly with increasing NaBH_4_ concentrations kinetic analyses revealed that Ni-POM offers equal or superior efficiency to other homogeneous catalysts with high conversion frequency (TOF) and low activation energy (*E*_a_) values. These results indicate that Ni-POM has strong potential for hydrogen production through the hydrolysis of NaBH_4_ and is a promising catalyst for clean energy production.

## Conflicts of interest

The authors declare that they have no known competing financial interests or personal relationships that could have appeared to influence the work reported in this paper.

## Data Availability

The data that support the findings of this study are available from the corresponding author upon reasonable request.
